# Dental Implant Failure Risk in Post Oncological Patients, a Retrospective Study and Sapienza Head and Neck Unit Decisional Protocol- 7 Years of Follow-Up

**DOI:** 10.3390/diagnostics12081863

**Published:** 2022-08-02

**Authors:** Edoardo Brauner, Valentino Valentini, Umberto Romeo, Marco Cantore, Federico Laudoni, Oriana Rajabtork Zadeh, Valeria Formisano, Andrea Cassoni, Marco Della Monaca, Andrea Battisti, Silvia Mezi, Alessio Cirillo, Francesca De Felice, Andrea Botticelli, Vincenzo Tombolini, Marco De Vincentiis, Andrea Colizza, Gianluca Tenore, Antonella Polimeni, Stefano Di Carlo

**Affiliations:** 1 Department of Oral and Maxillofacial Sciences, Sapienza University of Rome, Via Caserta 6, 00161 Rome, Italy; edoardo.brauner@uniroma1.it (E.B.); valentino.valentini@uniroma1.it (V.V.); umberto.romeo@uniroma1.it (U.R.); oriana.rajab@gmail.com (O.R.Z.); andrea.cassoni@uniroma1.it (A.C.); marco.dellamonaca@uniroma1.it (M.D.M.); andrea.battisti@uniroma1.it (A.B.); marco.devincentiis@uniroma1.it (M.D.V.); gianluca.tenore@uniroma1.it (G.T.); antonella.polimeni@uniroma1.it (A.P.); stefano.dicarlo@uniroma1.it (S.D.C.); 2 Independent Researcher, Corso Italia 19, 58015 Orbetello, Italy; 3 Independent Researcher, via Garibaldi 141, 00012 Guidonia, Italy; federico.laudoni@hotmail.it; 4 Independent Researcher, via Raffaele Cadorna 29, 00187 Rome, Italy; valeriaformisano5@gmail.com; 5 Department of Radiological Oncological and Pathological Science, Sapienza University of Rome, 00185 Rome, Italy; silvia.mezi@uniroma1.it (S.M.); alessio.cirillo@uniroma1.it (A.C.); francesca.defelice@uniroma1.it (F.D.F.); andrea.botticelli@uniroma1.it (A.B.); vincenzo.tombolini@uniroma1.it (V.T.); 6 Department of Sense Organs, Policlinico Umberto 1 Sapienza University of Rome, viale Regina Elena 326, 00161 Roma, Italy; andrea.colizza@uniroma1.it

**Keywords:** decisional protocol, postoncological patients, dental implant risk failure

## Abstract

(1) Background: Patients with head and neck cancer are treated by ablative surgery, radiotherapy, chemotherapy, or a combination of these. The side effects of cancer therapies can compromise conventional prosthesis rehabilitation; therefore, dental implants can result in a more effective solution. The aim of the study is to explain how to rehabilitate a patient that underwent head and neck cancer therapy. (2) Methods: This retrospective study conducted from 2015 to 2021 included 223 postoncological patients, aged between 32 and 80 years old. Eighteen patients did not proceed with any treatment, and two died. Therefore, 203 patients have been analyzed and rehabilitated following our decisional protocol, with a mean period of follow-up of 4 years. The implant placement was considered successful when a mean bone loss of 1.6 mm for the first year and a mean of 0.13 mm in subsequent years occurred (3) Results: A total of 161 patients were rehabilitated with a conventional prosthesis, 42 patients (F:M ratio 19:23) with an implant-supported prosthesis and a total of 200 implants were placed; 9 implants were lost (4.5% of 200 implants). Conclusions: The results confirmed that by following our protocol it is possible to obtain an acceptable rate of implant survival, considering the delicacy and complexity of post-oncological patients.

## 1. Introduction

The sixth most common cancer worldwide is head and neck cancer with an estimated global incidence of 830,000 new cases annually [1]. Patients with head and neck cancer are treated with ablative surgery, radiotherapy, chemotherapy, or a combination of these. Rehabilitating these patients is always a challenge, so it is important to define the best prosthetic solution [2]. Using conventional prostheses may not be the right choice, considering the changes in oral anatomy after ablative surgery and the side effects of cancer therapies, such as mucositis, xerostomia, and alterations of the bone healing processes [3]. Therefore, dental implants can potentially result in a more effective solution for oral rehabilitation. However, implant loss may occur more frequently in postoncological patients if the right precautions are not taken [4]. The aim of the present study is to propose a protocol, based on our experience, that allows establishing if a postoncological patient can proceed with implant-supported prosthesis rehabilitation, considering the implant failure risk factors typical of these patients.

## 2. Materials and Methods

This retrospective study is based on the data analysis of a total of 223 postoncological patients coming from the assistance activity carried out in the implant prosthesis department of Policlinico Umberto I from 2015 to 2021.

The inclusion criteria were: a previous clinical history of head and neck tumor treated with one or more cancer therapies (surgery, chemotherapy, radiotherapy).

The exclusion criteria were: all the patients that were not treated for head and neck cancer.

The study included the analysis of 223 patients, 18 did not want to proceed with any rehabilitation, 2 patients died. The patients have been under our observation from 2015 to 2021, the age ranged from 32–80 years old. The remaining 203 patients have been analyzed and then rehabilitated following our decisional protocol based on the implant failure risk assessment table (Table 1), allowing us to choose the right candidates for an implant-supported prosthesis rehabilitation. Chemotherapy or radiotherapy must be concluded before any oral rehabilitation. We considered the implant surgery successful when a mean bone loss of 1.6 mm for the first year and a mean bone loss of 0.13 mm in subsequent years occurred [5].

Table 1 elongates the different implant failure risk factor s, attributing to each one of them a color code that indicates the risk entity for implant failure.

### 2.1. Chemotherapy/Other Medical Therapy

Chemotherapy or other medical therapies that are related to osteonecrosis of the jaw (MRONJ) or in general may interfere with implant survival need to be investigated.

#### 2.1.1. Cytotoxic Drugs for Chemotherapy

Produce bone marrow suppression resulting in leukopenia by day 10 after the start of chemotherapy; thrombocytopenia after 10–14 days; anemia in a longer time frame [6]. All patients that have been on chemotherapy should be screened for bleeding risk. Coagulation profile, including platelet count, needs to be examined before starting any surgery or traumatic treatment. Elective dental surgery should be deferred in patients that received chemotherapy if the platelet count is <100,000/mm^3^ or if the patient has leucopenia of <1000/mm^3^ [7].

#### 2.1.2. Antiresorptive (AR) Drugs

##### Bisphosphonates

Bisphosphonates are divided into aminobisphosphonates (NBPs), including zoledronate, pamidronate, alendronate, risedronate, ibandronate, neridronate and non-aminobisphosphonates, such as clodronate, tiludronate, and etidronate. NBPs have greater affinity for bone, and a power ranging from 10 to 1000 times higher than BPs not containing amino groups [8].

Patients with osteometabolic disorders and with bone fragility, including CTIBL patients (cancer treatment-induced bone loss), that are treated with AR medications [9] have a considerably lower risk of developing MRONJ (1:100.000), with respect to patients with cancer receiving AR drugs for bone metastases or myeloma, because of the lower dosages prescribed. The incidence of ARONJ in oncologic patients receiving intravenous bisphosphonate therapies ranges from 0 to 12.2 per 100,000 patient-years [10]. The potency of the drugs utilized to treat bone metastases exceeds by a factor of 5–20 the oral drugs prescribed for osteometabolic disorders [11,12,13].

Patients who are currently on bisphosphonates and those who received them in the past need the same precautions and dental care, considering that the half-life of these drugs ranges from 1 to 10 years. The risk of ARONJ increases with the duration of the therapy [14].

##### Denosumab

Denosumab is a monoclonal antibody that works by forming immune complexes with RANK-L (Receptor Activator of Nuclear Factor kB-Ligand) inhibiting the recruitment and activation of osteoclasts in a temporary and reversible manner, with consequent reduction in bone turnover. Denosumab has two different trade names and two different methods of use: Xgeva for bone metastases and Prolia for osteoporosis. Patients with malignancies taking subcutaneous denosumab, have an AR incidence of 0 to 2316 per 100,000 patient-years [10]. If the patient is taking Denosumab for osteoporosis and oral interventions or needed the delay of the therapy it is not indicated due to the latency of administration every six months, it is sufficient to proceed with any dental procedure between the first and the third month after the drug administration in order to promote healing before the next administration.

#### 2.1.3. Other ONJ-Related Drugs (Not Prescribed for Head and Neck Cancer)

- Drugs with a prevalent anti-angiogenic action (bevacizumab e aflibercept).
- Tyrosine kinase inhibitors (sunitinib, sorafenib, cabozantinib, regorafenib, axitinib)m-TOR inhibitors (temsirolimus, everolimus).

For those patients that have been treated with AR drugs or in general with ONJ-related drugs it is important to determine the risk of MRONJ, establishing which drug was prescribed, the way of administration, the dosage, the duration of the therapy, and if there are any additional risk factors (chronic cortisone therapy; diabetes; smoking; rheumatoid arthritis or other connective tissue diseases). To evaluate the risk of MRONJ, we followed the protocol described in “A New Medical Record Proposal to the Prognostic Risk Assessment for MRONJ in Oncologic Patients: ≪Sapienza Head and Neck Unit≫ Proposal” [10]. This folder is based on the patient’s general condition, timing to start bone therapy and chemotherapy, and any condition that may increase the risk of MRONJ, and allows us to classify our patients, selecting the best oral treatment and follow-up plan for each one of them.

### 2.2. Radiotherapy

**Figure 1:**
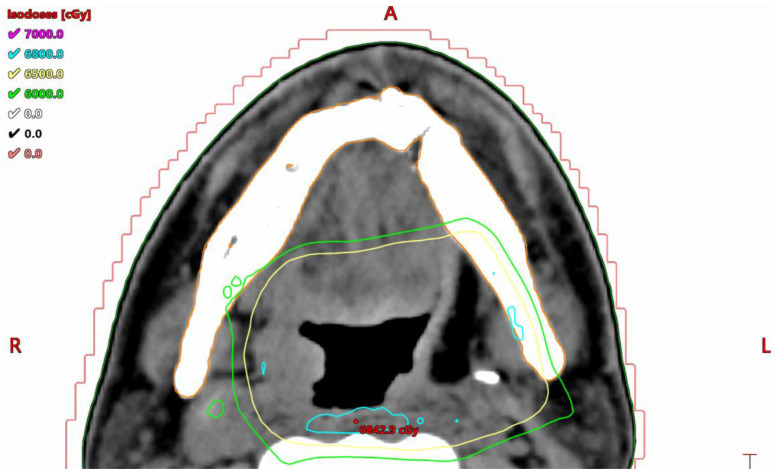
Absorbed dose distribution in mandible.

Nowadays, intensity-modulated radiotherapy (IMRT) is the technique of choice for HNC patients, due to its ability to highly conform dose coverage to the target while minimizing the high dose exposure to normal tissues. Acute and late toxicity is influenced by both treatment and patient-related parameters. Oral mucositis, xerostomia, dysphagia, and osteoradionecrosis represent the main side effects secondary to the irradiation of the oral cavity. In the case of implant procedure, in order to identify the most suitable sites for implant insertion and decrease the risk of ORN, it should be useful to adequately define the absorbed dose distribution of the mandible and jaw (Figure 1).

### 2.3. Bone Tissue Condition

There are systemic/local conditions that may interfere with bone healing or with osseointegration. 

#### 2.3.1. Systemic Factors

- Patient’s age:

In older patients, bone healing may be slower and failure rates may be slightly increased considering the changes that occur in the mineral composition of the bone. However, results from clinical investigations seem to reject this assumption [15,16,17,18].

- General health:

The overall patient’s health and the presence of general diseases influence the implant survival. Bone metabolic disease, (e.g., osteoporosis, osteomalacia, hyperparathyroidism, Paget’s disease), rheumatic diseases, (e.g., rheumatoid arthritis, Sjogren’s syndrome, systemic lupus erythematosus), hormonal diseases, (e.g., diabetes, Cushing’s syndrome, hyperparathyroidism), lichen planus, anomalies of neutrophil granulocytes, delayed hypersensitivity, immunological disorders, and malabsorption syndromes all influence the result of implant placement [19,20,21,22].

- Smoking:

Smoking not only is strongly correlated to oral cancer [23] but it is also recognized as an implant failure risk factor [24,25].

#### 2.3.2. Local Factors

- Bone quality, quantity, and anatomical location:

The characteristics of the bone and the anatomical site in which the implant will be inserted are factors that often influence implant survival. In general, the implant loss is more frequent in the maxilla rather than in the mandible, and in the posterior sectors compared to the anterior ones. Probably, the different bone quality and the different types of loads that the structures will have to support influence the implant survival [26,27,28,29,30,31,32,33,34,35,36,37,38,39,40,41,42].

**Figure 2:**
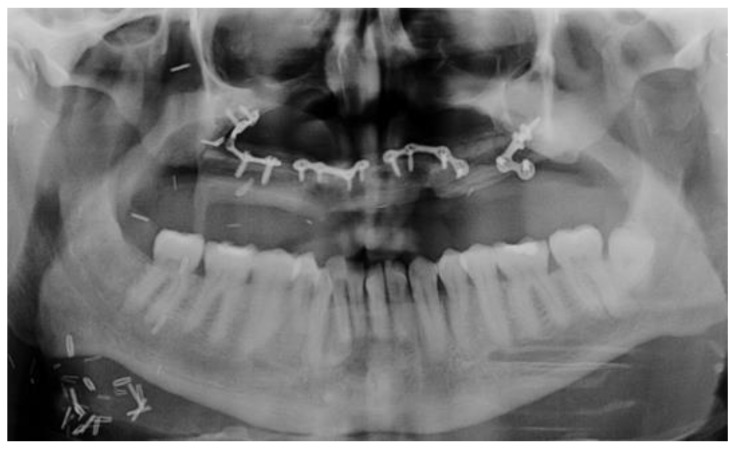
Patient after an upper jaw osteosarcoma resection and reconstruction with FFF. (19 December 2018).

**Figure 3:**
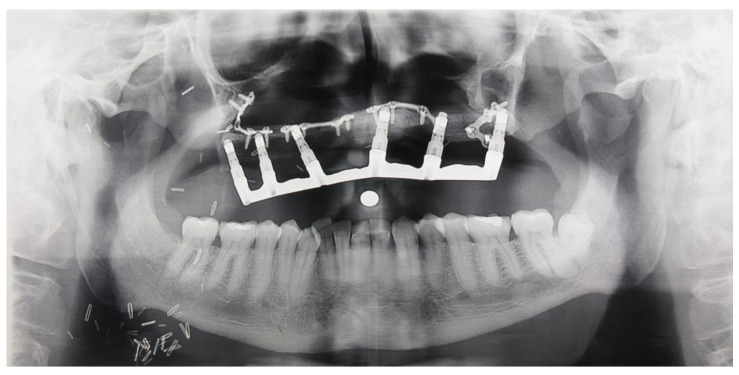
Final implant-supported prosthesis rehabilitation on a free fibula flap reconstruction. (25 February 2020).

Bone quality can be classified into four categories, based on its radiographic appearance and the resistance to drilling [43]: (1) “type 1 bone”, homogenous compact bone; (2) “type 2 bone”, dense trabecular bone surrounded by a thick cortical bone; (3) “type 3 bone”, in which a dense trabecular bone is surrounded by a thin layer of cortical bone; (4) “type 4 bone”, characterized by a core of low-density trabecular bone and a thin layer of cortical bone. Dental implants placed in grafted bone/free bone flaps bone can be considered as well performing as those placed in regular bone, as also described in the literature [44,45,46]. Figure 2 and Figure 3 present an example of free fibula flap (FFF) reconstruction and dental rehabilitation.

### 2.4. Soft Tissue Condition

Squamous cell carcinoma of the tongue is the most common intraoral malignancy, accounting for 26% of head and neck cancers [47,48]. Functional outcomes are affected by the extension and localization of tongue resection, type of reconstruction, residual tongue motility, and adjuvant radiotherapy [49,50,51,52].

#### 2.4.1. Tongue Mobility

**Figure 4:**
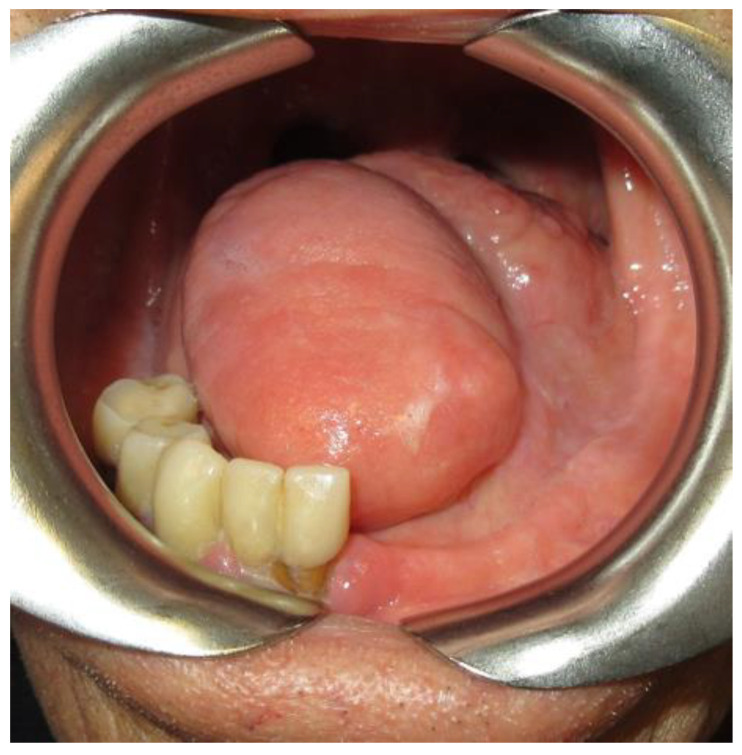
Hemiglossectomy.

When oral cancer involves the tongue or the nearby tissue, the surgical resection will inevitably affect the function of this muscle (Figure 4).

The tongue is fundamental for different functions, such as helping in the maintenance of oral hygiene by removing organic residues, clearing the buccal, labial, and alveolar sulci; in those patients that underwent surgery and have lost tongue mobility, it is frequent to observe inadequate oral hygiene resulting in decays formation or in the case of implants insertion in the risk of developing periimplantitis.

Different studies show that after reconstruction with either a forearm or thigh flap executed after hemiglossectomy, quite reasonable speech and swallowing functions are reobtained, although neither flap restores speech nor swallowing to the same level as that in normal individuals [53].

#### 2.4.2. Keratinized Gengiva

In periodontics, it has been suggested that the presence of an attached keratinized gingiva with a width of 2 mm or more is essential for periodontal health [54]. The adequate width of keratinized mucosa, around dental implants, that can guarantee optimal biological seal stability and resistance to mechanical forces has been discussed in the literature, [55].

For each of the above-listed implant failure risk factors (chemotherapy/other medical therapy, radiotherapy, bone, and soft tissue conditions) we established a color code (green, yellow, red), defining the possibility or not to proceed with an implant rehabilitation program.

##### Red Code

It indicates a high risk of implant failure. It is highly unrecommended to proceed with any traumatic intervention.

1. Chemotherapy: intravenous antiresorptive drugs assumption for bone metastases.
2. Radiotherapy: future prosthesis zone directly irradiated with a total dose ≥66 Gy (2 Gy/fraction).
3. Bone tissue condition: insufficient bone volume, the impossibility of bone augmentation for implant insertion, presence of jaw plates.

##### Yellow Code

It indicates a moderate risk of implant failure. If multiple yellow codes are identified, it is important to consider the overall patient’s condition. Whenever the yellow color code is identified it is necessary to take precautions and deepen the topic.

1. Adjuvant Chemotherapy/antiresorptive (AR) or antiangiogenic (AA) therapy

If the patient underwent antiangiogenic therapy, it is necessary to prescribe blood exams for platelets and leukocyte count. Elective dental surgery should be deferred in patients that received chemotherapy if the platelet count is <100,000/mm^3^ or if the patient has leucopenia of <1000/mm^3^.

If AR drugs were administrated, it is necessary to define.

- The medical therapy specifies (drug type, dosage, the way of administration, and period of intake).
- The specifics of any additional pharmacological therapy (cortisone, anticoagulative, anti-aggregating drugs).
- The presence of any pathology that may interfere with the healing processes of the bone.

Although adjuvant chemotherapy or osteometabolic disorders therapies do not preclude traumatic interventions such as implant insertion, it is opportune to consider the overall case, the patients’ health, and the presence of eventual comorbidities.

2. Radiotherapy

Based on dose distribution it is possible to define the precise irradiation zone and received dose. If the total dose is between 50 and 64 Gy (2 Gy/fraction) then it is possible to proceed with relative caution, always considering the dose distribution and the overall patient’s health.

3. Bone tissue condition

Reduced bone volume or unfavorable bone healing conditions. An adequate bone volume is required for implant placement. We considered to be sufficient for implant insertion:

- A bone height of at least 8 mm.
- A bone thickness that allows keeping at least 1 mm of bone around the implant itself.
- A healthy and good quality tissue.

4. Soft Tissue Condition.

The absence of a low quantity of keratinized gingiva (less than 2 mm of width) or reduced tongue mobility both represent risk factors for mucosa inflammation and implant periimplantitis and must be considered in the overall treatment plan.

##### Green Code

It indicates low risk for implant failure

1. No chemotherapy.
2. No radiotherapy or a dose <50 Gy received by the future implant zone. Attention should be paid to obtain an appropriate result even in case of dose <50 Gy.
3. Bone tissue: healthy tissue; sufficient bone volume. Reconstructed bone or grafted bone as in the case of a free fibula flap has been demonstrated to be a good bone for implant insertion [56,57,58].

Soft tissue: sufficient keratinized gingiva width (more than 2 mm around the implant) and no affections of tongue mobility.

## 3. Results

**Scheme 1:**
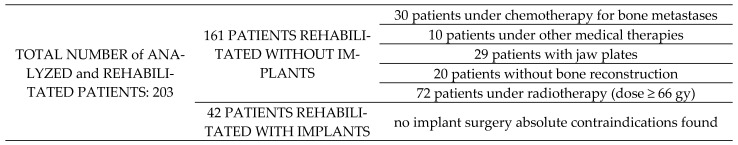
Patients rehabilitated with or without implants.

From a total of 223 postoncological patients, 18 did not accept to proceed with any treatment, and 2 died. Therefore, 203 patients have been analyzed and then rehabilitated (Scheme 1) following our decisional protocol based on the implant failure risk assessment table (Table 1). A total of 161 patients were rehabilitated with a non-implant supported prosthesis because the requirements for surgery were not respected: 72 patients received more than 64 Gy (2 Gy/fraction) at the future implant site; 30 were treated with chemotherapy for bone metastases, and 10 were under concomitant medical therapies so implant surgery was precluded; 29 had jaw plates; hence, it was not possible to insert any implant; 20 did not receive any bone reconstruction after the tumor resection.

A total of 42 patients (F: M ratio of 19:23) were selected for implant rehabilitation (Table 2). These 42 patients underwent different cancer treatments: 30 underwent only resective surgery; 5 surgery + adjuvant radiotherapy (total dose 60–66 GY, 2 Gy/fraction); 4 surgery + neoadjuvant chemotherapy; 3 had surgery + adjuvant chemo-radiotherapy (total dose of 66 Gy, 2 Gy/fraction). 8 patients received soft tissue reconstructions: 5 with an anterolateral thigh muscle (ALT) flap; 3 with a pedunculated temporal muscle flap; 1 with a pectoralis major myocutaneous flap.

13 bone defects were reconstructed: 7 with a free fibula flap, and two of these patients, who did not undergo radiotherapy, also received guided bone regeneration surgery; 5 with a free iliac crest flap; 1 with a free scapular flap. 21 did not receive any reconstructive surgery.

A total of 200 implants were inserted and subjected to follow-up from 1 to 8 years. A total of nine implants were lost (four in the early phase, five after a year from insertion). Three were lost in patients treated with radiotherapy. The implant success was calculated considering as successful a mean bone loss of 1.6 mm for the first year and a mean of 0.13 mm in subsequent years (5). The total implant failure rate obtained was 4.5%.

Scheme 1 explains why 161 patients were rehabilitated without implants and why 42 patients were selected to proceed with an implant supported prosthesis rehabilitation. 

## 4. Discussion

Postoncological patients affected by head and neck cancer present unfavorable outcomes derived from cancer therapy side effects. Patients with head and neck cancer that have been treated with ablative surgery, suffer from severe post-surgery anatomy alterations, interfering with the stability of the removable prosthesis. Therefore, bone graft reconstructions and implant-supported prostheses may represent the best solution for these patients.

It has been demonstrated by J.P. Hayter et al. [46], that endosteal implants inserted in a free flap reconstruction are a great solution for patients that underwent drastic surgery. However, implant placement in grafted bones is not free from complications, in fact, a frequent problem that occurs after reconstruction with free vascularized flaps is the onset of hyperplastic granulomatous reactive tissue around the prosthetic abutment of the implant, even though the implant survival does not seem to be significantly affected as concluded by Brauner et al. in their study [59].

Soft tissues are also involved in cancer treatments. The eventual resection of salivary glands causes xerostomia and changes in salivary fluid and radiotherapy may cause xerostomia, mucositis, and alterations in healing processes. The lack of wetting of the oral mucosa would result in chronic irritation, the insurgence of ulcerations, or areas of bone exposure with the impossibility to realize a functional prosthesis.

Chemotherapy or other medical therapies with AR or AA drugs generate biological changes that influence healing processes and that increase the risk of developing severe oral complications.

Cycles of adjuvant or neoadjuvant chemotherapy are correlated to oral complications as explained by Dreizen et al. [6].

One of the most discussed oral complications is bisphosphonate-related osteonecrosis of the jaw (BRONJ), The risk factors and the possible preventive treatments, indicate the best way to approach patients that received or are still undergoing bisphosphonate therapy are explained by Ruggiero et al. [7].

For those patients who underwent chemotherapy with antiresorptive or antiangiogenics drugs, for bone metastasis, it is not recommended to proceed with implant placement, because the high dosages and the intravenously way of administration represent high-risk factors for implant failure as concluded in the study conducted by Brauner et al. [10].

On the other side, patients treated with chemotherapy or other drug therapies not for bone metastases can be evaluated for future dental implant rehabilitation, but precautions need to be taken. The sample must increase its number to collect larger amounts of data as patients who have undergone chemotherapy represent the 16.6% of the total number of patients in which the implants were placed.

Radiotherapy represents another obstacle for the postoncological patient’s oral rehabilitation, in fact, the risk of implant failure increases when implants are placed in the irradiated bone as De la Plata et al. affirmed [3]. Oral cavity irradiation can cause a reduction in bone vascularization, clinically manifested as osteoradionecrosis, hindering the osseointegration of the implants.

There is much variation in the reported success rates of implant insertion. De La Plata et al. [3] obtained a 5-year survival rate of 92.6% in irradiated patients, highlighting that irradiated patients had a significantly higher rate of implant loss than non-irradiated patients. At 1, 5, and 10 years of follow-up, an implant success rate of over 89% was reported in irradiated bone by Linsen et al. [60]. Different radiation techniques have been demonstrated to not affect the outcomes of implant rehabilitation, as demonstrated by Papi et al. [61].

The results of our retrospective study revealed that 33% of the lost implants occurred in patients who received radiotherapy. Traditionally, radiotherapy for head and neck cancer is delivered with an IMRT technique with a total dose between 50 and 70 Gy (2 Gy/fraction). IMRT allows for the evaluation of the absorbed dose distribution of the bone. Therefore, in the evaluation of irradiated patients, it is important to carefully examine which zones have been irradiated, the fractionation, and the overall treatment time, by using the data provided by the dose–volume histogram (DVH) as explained by Brauner et al. [62]. Since IMRT is available and the study of the irradiated zone is much more precise, we have noticed a significant reduction in failed implants.

Considering all the side effects of the different cancer therapies, the right rehabilitation for postoncological patients is usually an implant-supported prosthesis, and the implant failure risk assessment table allows to establish if it is possible or not to proceed with implant insertion. By analyzing the items in Table 1, chemotherapy/other medical therapies, radiotherapy, bone, and soft tissue conditions, it is possible to define the risk of implant loss for each patient.

All this information can provide a detailed picture of the case and decrease the risk of losing implants.

By following our protocol and by using the implant failure risk assessment table, it is possible for the clinician to select the best rehabilitation solution for postoncological patients. It is important to consider that if the patient presents more risk factors simultaneously then the risk of implant failure will be higher and more unpredictable. Even so, the results demonstrate that by following this protocol the implant failure rate obtained was 4.5%.

## 5. Conclusions

In patients undergoing cancer treatments aimed at tumor elimination such as surgery, radiotherapy, and chemotherapy and subsequently treated or not with regenerative surgical methods, rehabilitation with removable prostheses is not elective rehabilitation therapy. To increase the quality of life of these patients, implant-supported rehabilitation is the first choice whenever possible. Implant insertion for postoncological patients needs to be carefully evaluated considering the patient’s general health and the conditions that may interfere with implant survival. In the literature, they are not many studies that explain how to approach this kind of patient. From the results obtained in our study, it is clear that the side effects of radiotherapy are still really dangerous for implant success, in fact, three of the nine lost implants were lost in patients that underwent radiotherapy. Therefore, it is important to consider all the risk factors that a postoncological patient presents, but it is also fundamental to not consider separately these factors and comprehend how these factors define the general patient’s health. The implant failure risk assessment table is a tool that guides the clinician to the best rehabilitative solution. Our operating protocol in the choice of patients who can undergo implant placement guarantees to reduce implant failure in postoncological patients, in our case we obtained a 4.5% implant failure rate. It is important to continue the evaluation, to increase the patient’s number, to increase the follow-up period, and to understand even more how all the risk factors condition the patient’s health and the risk of implant failure.

## Figures and Tables

**Table 1:** Implant failure risk assessment table.

Implant Failure Risk Factors		Risk Entity
**CHEMOTHERAPY or OTHER MEDICAL THERAPY**	FOR BONE METASTASES	
	ADJUVANT CHEMOTHERAPY	
	OSTEOMETABOLIC DRUG THERAPY	
	NO CHEMOTHERAPY OR OTHER DRUG THERAPY	
**RADIOTHERAPY (dose at the implant zone)**	DOSE ≥ 66 Gy	
	50 Gy ≥ DOSE < 66 Gy	
	DOSE < 50 Gy	
	NO RADIOTHERAPY	
**BONE TISSUE**	NO BONE PRESENT/JAW PLATES PRESENT	
	PARTIALLY PRESENT OR UNFAVORABLE SISTEMIC/LOCAL CONDITIONS	
	PRESENT	
	RECONSTRUCTED BONE	
**SOFT TISSUE**	LOW TONGUE MOBILITY	
	DEFICIENCY/ABSENCE OF KERATINIZED GENGIVA IN FUTURE IMPLANT INSERTION ZONE	
	SUFFICIENT KERATINIZED GENGIVA IN FUTURE IMPLANT INSERTION ZONE	
	NORMAL TONGUE MOBILITY	
**LEGEND**		
HIGH RISK OF IMPLANT FAILURE		
MODERATE RISK OF IMPLANT FAILURE		
LOW RISK OF IMPLANT FAILURE		

**Table 2:** Patients rehabilitated with implants.

Total Number of Patients Rehabilitated with Implants	Total Number of Insterted Implants	Total Number of Lost Implants	% of Lost Implants
42	200	9	4.5%
